# Impact of G-Quadruplex Structures on Methylation of Model Substrates by DNA Methyltransferase Dnmt3a

**DOI:** 10.3390/ijms231810226

**Published:** 2022-09-06

**Authors:** Andrei G. Loiko, Alexander V. Sergeev, Adelya I. Genatullina, Mayya V. Monakhova, Elena A. Kubareva, Nina G. Dolinnaya, Elizaveta S. Gromova

**Affiliations:** 1 Department of Chemistry, Lomonosov Moscow State University, Leninskie Gory 1, 119991 Moscow, Russia; 2 Belozersky Institute of Physico-Chemical Biology, Lomonosov Moscow State University, Leninskie Gory 1, 119991 Moscow, Russia

**Keywords:** G-quadruplex, Dnmt3a, DNA methylation, protein–DNA binding

## Abstract

In mammals, de novo methylation of cytosines in DNA CpG sites is performed by DNA methyltransferase Dnmt3a. Changes in the methylation status of CpG islands are critical for gene regulation and for the progression of some cancers. Recently, the potential involvement of DNA G-quadruplexes (G4s) in methylation control has been found. Here, we provide evidence for a link between G4 formation and the function of murine DNA methyltransferase Dnmt3a and its individual domains. As DNA models, we used (i) an isolated G4 formed by oligonucleotide capable of folding into parallel quadruplex and (ii) the same G4 inserted into a double-stranded DNA bearing several CpG sites. Using electrophoretic mobility shift and fluorescence polarization assays, we showed that the Dnmt3a catalytic domain (Dnmt3a-CD), in contrast to regulatory PWWP domain, effectively binds the G4 structure formed in both DNA models. The G4-forming oligonucleotide displaced the DNA substrate from its complex with Dnmt3a-CD, resulting in a dramatic suppression of the enzyme activity. In addition, a direct impact of G4 inserted into the DNA duplex on the methylation of a specific CpG site was revealed. Possible mechanisms of G4-mediated epigenetic regulation may include Dnmt3a sequestration at G4 and/or disruption of Dnmt3a oligomerization on the DNA surface.

## 1. Introduction

Methylation of cytosine residues at CpG sites is a basic epigenetic DNA modification involved in the regulation of gene expression, maintenance of genomic stability, aging, and other biological processes. In mammals, cytosine methylation at the C5 is installed and maintained by three DNA methyltransferases (MTases), Dnmt1, Dnmt3a, and Dnmt3b; de novo DNA methylation, i.e., the implementation of a methylation pattern (a kind of alternation of methylated and unmethylated CpG sites), is performed by Dnmt3a [1,2]. The promoter regions of actively transcribed genes containing CpG islands are usually unmethylated, with oncogene promoters tending to be highly methylated and promoters of tumor suppression genes being largely depleted of methylation [3]. The abnormal methylation status has been shown to have severe consequences for the organism. Recently, a key role of Dnmt3a in the onset and progression of several types of cancers has been reported; in malignant tumor cells, Dnmt3a overexpression, mutations in the *Dnmt3a* gene, and an aberrant methylation pattern are observed [4,5,6].

The control of DNA methylation level is critical to gene regulation, and the factors that drive hypomethylation at CpG islands are still being uncovered. One such factor may be the formation of DNA G-quadruplexes (G4s) near methylation sites. The presence of G4s, the most surprising and widely studied noncanonical forms of DNA, in promoters of some genes, especially in many oncogene promoters, has been rigorously proven [7,8,9,10,11,12,13]. Endogenous G4s are found in certain G-rich sequences (G4 motifs) and arise through the self-association of guanine residues to form stacked G-tetrads, which are stabilized by Hoogsteen hydrogen bonds and by interactions with metal ions (mainly K^+^) that are coordinated in the central cavity. The G4 structures are highly polymorphic; they can adopt parallel, antiparallel and hybrid (3 + 1) topologies characterized by different orientation of the four G-tracts in the quadruplex core [12,14]. Being structural elements of the genome, G4s are recognized by numerous cellular proteins and enzymes and interfere with basic cellular processes, such as DNA replication, transcription, chromosome end protection, mutagenesis, DNA repair, and recombination [15]. However, there are few publications on the involvement of G4 structures in the functioning of the DNA methylation machinery [16].

In 2016, Cree et al. reported high affinity binding of human recombinant MTases to DNA G4s formed in promoters of human genes such as *CDKN1C*, *c-MYC*, and others using surface plasmon resonance spectroscopy [17]. In this work, G4 structures were modeled by G4-forming oligonucleotides, and binding constants were comparable to those of many cellular proteins known to recognize G4 structures in a genomic context. A recently published study [18] addressed a new era in this field, namely the interplay between G4 formation and hypomethylation of certain DNA regions. The DNMT1 methylation activity has been shown to be inhibited by G4 structures. The authors documented the ability of maintenance DNMT1 MTase to colocalize at sites of G4 formation and proposed a mechanism for protecting CpG islands from methylation by G4 structures that locally sequester and inhibit DNMT1. However, a number of issues are still being uncovered. MTase functioning was examined using isolated G4s but not the G4 structure embedded in the DNA double helix. The G4 impact on de novo methylation by Dnmt3a has not been studied at all; the effect of G4-forming oligonucleotides has only been described for maintenance DNMT1 via inhibition of methylation. Finally, the participation of individual MTase domains was not taken into account, although these domains are responsible for different MTase functions: chromatin targeting or direct involvement in the methylation event.

The aim of this work was to establish the relation between the G4 formation in DNA and the functioning of the murine MTase Dnmt3a or its individual domains in vitro. The DNA substrates were modeled with synthetic oligonucleotides containing the d(GGGT)_4_ sequence capable of folding into the well-characterized parallel G4 structure inherent in the promoter G4s. This G4 motif was either embedded in a single-stranded oligonucleotide or stabilized in a DNA duplex context containing several CpG sites.

## 2. Results

### 2.1. Selection and Design of Proteins and DNA Substrates

**Figure 1:**
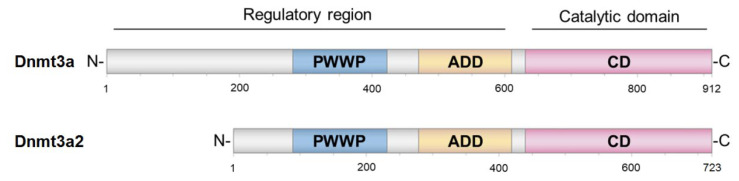
Domain architecture of full-length MTase Dnmt3a and its isoform Dnmt3a2. ADD indicates ATRX-DNMT3-DNMT3L domain; PWWP is Pro-Trp-Trp-Pro domain.

The active form of eukaryotic MTase Dnmt3a is a linear heterotetramer containing two active sites at a distance corresponding to 8–14 bp of DNA [1,19]. It is believed that the enzyme tetramer is capable of oligomerization on DNA [20]. Dnmt3a contains a C-terminal catalytic domain (Dnmt3a-CD) and an N-terminal regulatory region (Figure 1).

The regulatory region contains two main domains, PWWP and ADD, responsible for interaction with chromatin and a wide variety of proteins (Figure 1) [21]. PWWP is responsible for heterochromatin targeting through the binding of its hydrophobic pocket to the nucleosomal histone H3 with lysine 36 bearing a di- or trimethylation mark (H3K36me2/3) [22,23]. The ADD domain specifically recognizes and binds to unmethylated lysine 4 of histone H3 (H3K4me0), mediates interactions with various protein factors, and is involved in the allosteric control of DNMT3A MTase activity [22].

To explore the potential relationship between G4 and methylation levels, we employed the murine Dnmt3a isoform, Dnmt3a2. Dnmt3a2 has a shortened regulatory region: the region spanning amino acids (aa) 1–212 is replaced by other 24 aa; the rest of the molecule shares 98% sequence identity with human DNMT3A [24]. Dnmt3a2 exists in an autoinhibitory conformation because the ADD domain blocks the DNA binding site of the catalytic domain [25]. To remove the autoinhibition of Dnmt3a2, substitutions Y526A and Y528A were simultaneously introduced into the ADD domain resulting in AIR-Dnmt3a2. In what follows, we refer to it as a full-length MTase, since all key domains have been derived from this form of enzyme. In addition to the AIR-Dnmt3a2, Dnmt3a-CD as well as the PWWP domain from the regulatory region of Dnmt3a were also selected and studied. Dnmt3a-CD can catalyze the DNA methylation reaction without the regulatory region [21]. Besides the ability of PWWP to bind H3K36me2/3, this domain is able to interact with nucleosomal DNA [26]. Dnmt3a-CD recognizes CpG-containing sequences, whereas PWWP is not specific to CpG-containing DNA duplexes. The prokaryotic CpG-recognizing MTase SssI (M.SssI) [27] was also assessed.

**Figure 2:**
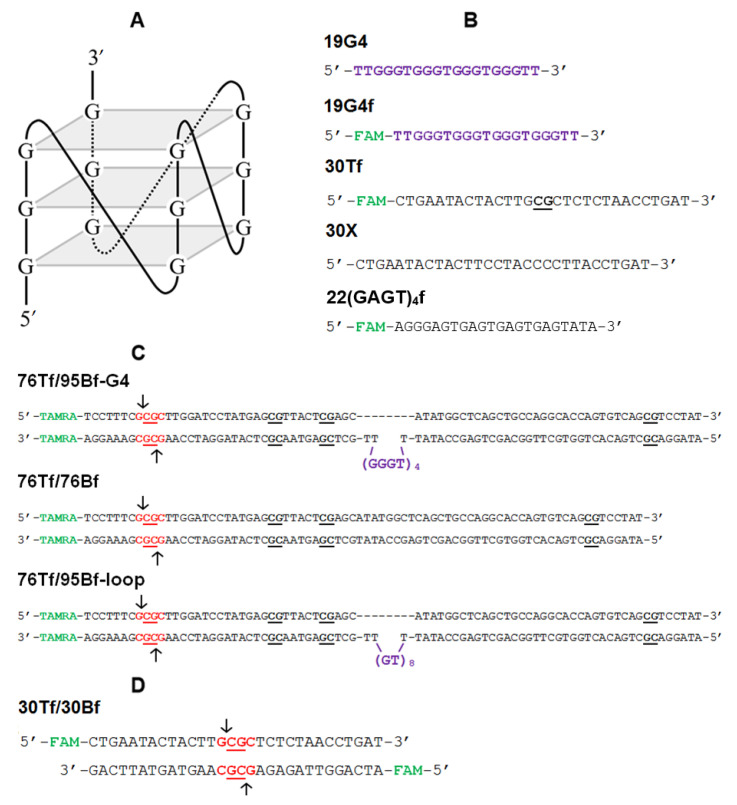
Abbreviation and sequence of oligonucleotides and DNA duplexes tested in this work. (**A**) Schematic representation of the parallel G4 structure formed by the d(GGGT)_4_ sequence. (**B**) 19-nt oligonucleotides capable of forming the G4 structure as well as single-stranded DNA controls. (**C**) The DNA duplex in which the G4 structure was stabilized in a double-stranded context; 76 bp perfect DNA duplex and DNA duplex with looped out d(GT)_8_ moiety used as double-stranded controls. (**D**) The 30 bp DNA duplex used as a known MTase substrate. CpG sites are highlighted in bold and are underlined; arrows indicate the sites of cleavage by restriction endonuclease Hin6I (R.Hin6I) (its recognition site is highlighted in red: 5′-G↓**CG**C-3′); TAMRA and FAM fluorophores are indicated in green.

Synthetic DNA models included: (i) oligonucleotides 19G4 and 19G4f, capable of folding into a parallel G4 structure (Figure 2A,B), as well as control randomized oligonucleotides 30Tf and 30X, which differ in the presence of a CpG site, and 22(GAGT)_4_f oligonucleotide containing four G > A substitutions compared to 19G4f, which prevent the G4 formation (Figure 2B); (ii) a DNA duplex containing a G4-forming insert (76Tf/95Bf-G4), as well as a control perfect duplex 76Tf/76B and a double-stranded DNA containing a looped out d(GT)_8_ sequence (76Tf/95Bf-loop) (Figure 2C); (iii) DNA duplex 30Tf/30Bf used as a known MTase substrate (Figure 2D). In the designation of DNA models, the following points are indicated: the oligonucleotide length, the strand location—conditionally top or bottom (T or B, respectively), the presence of a fluorescent label (f), 5-carboxytetramethylrhodamine (TAMRA) or 6-carboxyfluorescein (FAM), and the embedded G4 motif (G4) or unstructured d(GT)_8_ repeat (loop). In our DNA models, the biologically relevant d(GGGT)_4_ sequence, known for its wide distribution in eukaryotic genomes and strong ability to form a stable G4 with a parallel topology inherent in promoter G4s, was applied. As DNA duplexes containing G4 or GT repeats, we used the original DNA constructs proposed in our previous paper [28], in which the G4 or loop structures were stabilized in a double-stranded context. To prevent the formation of a competing Watson–Crick double helix, these DNA models were prepared by hybridization of partly complementary strands, one of which contained the G4 motif or d(GT)_8_ repeat flanked by oligonucleotide fragments, while the opposite strand lacked a site complementary to oligonucleotide inserts; the sequence of the DNA duplex flanks was chosen arbitrarily.

The secondary structure of the engineered 76Tf/95Bf-G4 and 76Tf/95Bf-loop models as well as the G4 folding topology and the thermal stability of both the G4 and duplex domains were earlier analyzed by a variety of biophysical and biochemical techniques [28]. The quadruplex folding capacity of 19G4 was evaluated in this work by spectroscopic methods (see below).

Of note, the double-stranded regions, flanking upstream and downstream the G4 structure in 76Tf/95Bf-G4 DNA model, contain several CpG sites. One of them was introduced into the recognition site of R.Hin6I to enable our method for determining the methylation activity of eukaryotic MTases [29]. The model DNA duplexes were annealed in buffer A containing 100 mM KCl unless otherwise specified. This concentration of potassium ions favors both G4 and B-DNA formation and is close to intracellular conditions.

### 2.2. CD and UV Spectroscopy Confirm the Existence of a Parallel G4 Structure Folded within 19G4

**Figure 3:**
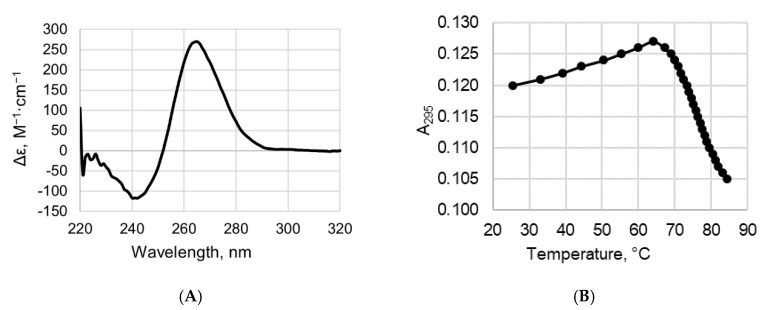
Analysis of the G4 structure formed by the oligonucleotide 19G4 by spectroscopic methods. (**A**) CD spectrum recorded in buffer A containing 100 mM KCl at 25 °C (~2 µM oligonucleotide concentration). (**B**) Thermal melting curve measured at 295 nm in buffer B containing 2.5 mM KCl at ~4 µM oligonucleotide strand concentration.

We applied a combination of circular dichroism (CD) and ultraviolet (UV) spectroscopy to study the secondary structure and thermal stability of the DNA quadruplex formed by 19G4. CD spectra of 19G4 were examined in buffer A containing 100 mM KCl; this K^+^ concentration close to that in human cells (~150 mM KCl) was used in our G4-protein binding experiments and MTase-mediated methylation analysis. The CD spectrum of 19G4 recorded at room temperature revealed a typical parallel G4 fold with a positive peak at 265 nm and a negative one around 240 nm (Figure 3A) [30]. Using CD data recorded at different temperatures, we obtained the 19G4 melting curve (Supplementary Figure S1, inset), but due to the high thermal stability of quadruplex structure in the presence of 100 mM KCl, an accurate assessment of the 19G4 melting temperature (T_m_) was not possible. For this reason, the temperature dependence of UV absorbance at 295 nm, which is a marker of a G4 structure, was measured in buffer B containing 2.5 mM KCl; a reduced KCl concentration was chosen to capture the entire G4-coil conformation transition within the available temperature range (Figure 3B). The hypochromic effect and a sigmoidal shape of the melting profile indicate the G4 unfolding with a T_m_ of 76 ± 1 °C. Spectroscopic data confirm that the d(GGGT)_4_ sequence, flanked by d(TT) dinucleotides, folds into an extremely stable parallel G4 even under potassium-depleted conditions.

### 2.3. Binding Affinity of AIR-Dnmt3a2 and Its Domains to DNA Substrates Containing the G4 Structure

#### 2.3.1. Electrophoretic Mobility Shift Assay (EMSA)

**Figure 4:**
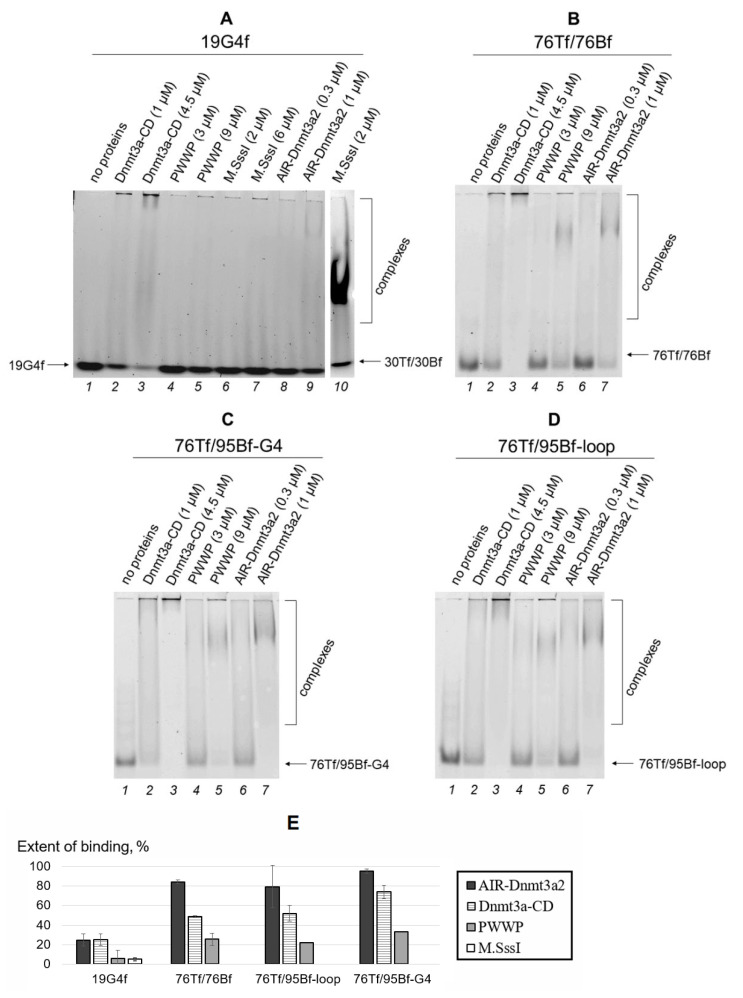
Binding affinity of full-length AIR-Dnmt3a2 and its domains to various DNA models using EMSA. (**A**–**D**) Protein•DNA complex formation recorded by electrophoresis in 3% non-denaturing polyacrylamide gel. The zones of protein•DNA complexes are indicated by parenthesis; arrows on the right indicate the position of unbound DNA. (**A**) 19G4f (lanes 1–9) or 30Tf/30Bf (lane 10), (**B**) 76Tf/76Bf, (**C**) 76Tf/95Bf-G4, and (**D**) 76Tf/95Bf-loop (60 nM) were incubated for 30 min in buffer A with AIR-Dnmt3a2, Dnmt3a-CD, PWWP, or M.SssI. (**E**) Extent of DNA binding to AIR-Dnmt3a2 (1 μM), Dnmt3a-CD (1 μM), PWWP (3 μM), or M.SssI (2 μM) calculated from the EMSA data. Standard deviations of two independent measurements are indicated.

The designed DNA models, including those containing a parallel-stranded intramolecular G4s, were used to address whether full-length AIR-Dnmt3a2 and its catalytic and regulatory domains (Dnmt3a-CD and PWWP, respectively) bind directly to G4 structures. Complex formation between 60 nM DNA labeled with a fluorophore and the proteins at various concentrations was monitored by EMSA. A series of measurements was carried out after the incubation of components in the presence of 100 μM *S*-adenosyl-l-homocysteine (AdoHcy), an analog of a cofactor *S*-adenosyl-l-methionine (AdoMet), which promotes assembly of the specific MTase•DNA complex [31]. The resulting gels are shown in Figure 4A–D. The bands on the gel correspond to unbound DNA (fast migrating bands) and protein•DNA complexes (slow migrating ones). Dnmt3a-CD is prone to oligomerization [21], and its complexes with DNA partially failed to enter the non-denaturing 3% polyacrylamide gel (Figure 4A–D, lanes 2, 3). Band intensities were used to estimate the percentage of protein•DNA complexes formed relative to total DNA (Section 4.5.1 and Figure 4E). 

We first used a simple model in the form of an isolated G4, 19G4f, to study the binding affinity of full-length AIR-Dnmt3a2 and its domains to this noncanonical DNA structure. At the same protein concentration (1 μM), the extent of 19G4f binding was around 25% for both AIR-Dnmt3a2 and Dnmt3a-CD, while the binding affinity for the regulatory PWWP domain, even at a threefold increase in concentration, proved to be less than 10% (Figure 4A,E). Of note, as compared to Dnmt3a-CD, the prokaryotic MTase M.SssI, which has a high methylation activity and can efficiently bind to the cognate DNA substrate, 30Tf/30Bf, forms a much weaker complex with 19G4f (Figure 4A, lanes 6, 7, and 10; Figure 4E).

It was important to compare the binding affinity of 19G4f to AIR-Dnmt3a2 and Dnmt3a-CD with that of the control oligonucleotide 22(GAGT)_4_f, which is of similar length but fails to form a G4 structure due to four G > A substitutions in the d(GGGT)_4_ quadruplex motif (Figure 2B). Compared to 19G4f, 22(GAGT)_4_f showed a four-times weaker binding affinity to AIR-Dnmt3a2 and Dnmt3a-CD without any notable difference between the full-length enzyme and its catalytic domain (Supplementary Figure S2). Therefore, these data highlight the high affinity of Dnmt3a to G4 structure.

We then characterized the binding affinity of AIR-Dnmt3a2 and its domains to the G4 structure stabilized in the duplex surrounding (76Tf/95Bf-G4) (Figure 4C,E) compared to a similar model with a non-structured d(GT)_8_ loop (76Tf/95Bf-loop) (Figure 4D,E) and the perfect DNA duplex 76Tf/76Bf (Figure 4B,E). It was revealed that 1 μM AIR-Dnmt3a2 almost quantitatively binds 76Tf/95Bf-G4. However, the binding efficiency of Dnmt3a-CD (1 μM) decreases to ~70%, while for the PWWP domain (3 μM) it reaches only ~30% (Figure 4C,E). A similar pattern of changes in the binding extent was observed for the loop-containing DNA duplex (76Tf/95Bf-loop) and the perfect duplex 76Tf/76Bf (Figure 4E). PWWP could effectively bind DNA duplexes only at a protein concentration of 9 µM (Figure 4B–D). The G4-containing substrate 76Tf/95Bf-G4 showed a significant advantage in binding to Dnmt3a-CD compared to the 76Tf/95Bf-loop and 76Tf/76Bf (Figure 4E). The last two DNA duplexes bound Dnmt3a-CD with equal efficiency. We found no difference in the binding of the studied DNA duplexes to AIR-Dnmt3a2 or PWWP. 

The results obtained were confirmed by an alternative calculation of protein•DNA binding efficiency derived from the EMSA data (Figure 4A–D). This approach was based on the decrease in the fluorescence intensity of the bands corresponding to unbound DNA upon the addition of the proteins (Supplementary Figure S3).

#### 2.3.2. Fluorescence Polarization Assay

**Figure 5:**
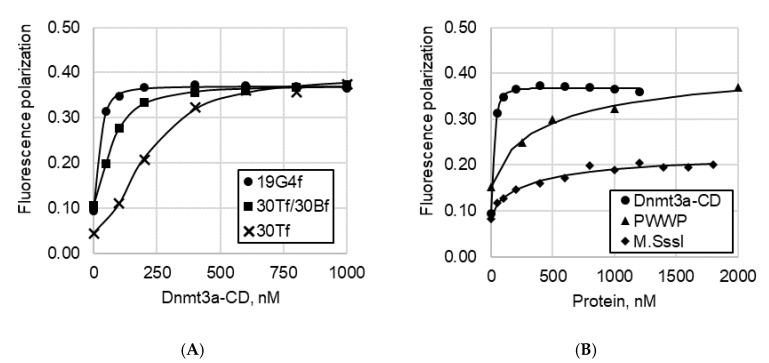
Curves of DNA binding to Dnmt3a domains and prokaryotic MTase M.SssI obtained from the fluorescence polarization assay data. (**A**) Dnmt3a-CD binding to FAM-labeled 19G4f, 30Tf, or 30Tf/30Bf. (**B**) 19G4f binding to Dnmt3a-CD, PWWP domain, or M.SssI. DNA probes (10 nM) were incubated with proteins in buffer A in the presence of 100 µM AdoHcy.

To quantitatively characterize the binding parameters of DNA substrates containing G4 inserts to Dnmt3a domains in solution, fluorescence polarization assay was used as an independent method. In the first series of experiments, direct titration of fluorescently labeled DNAs with Dnmt3a-CD or PWWP was performed. The curves of protein binding to DNA substrates are presented in Figure 5, and the corresponding dissociation constant (*K_d_*) values are in Table 1. According to data obtained, the G4-containing oligonucleotide 19G4f binds to Dnmt3a-CD with a low nanomolar affinity, which is seven and three times higher than that of the known Dnmt3a double-stranded substrate, 30Tf/30Bf, lacking the G4 structure, and its top oligonucleotide strand, 30Tf, respectively. Notably, 19G4f showed poor binding to the PWWP domain of Dnmt3a and to the prokaryotic MTase M.SssI (Figure 5B). The *K_d_* values for these complexes exceeded those for Dnmt3a-CD•19G4f by factors of 13 and 11, respectively (Table 1).

**Figure 6:**
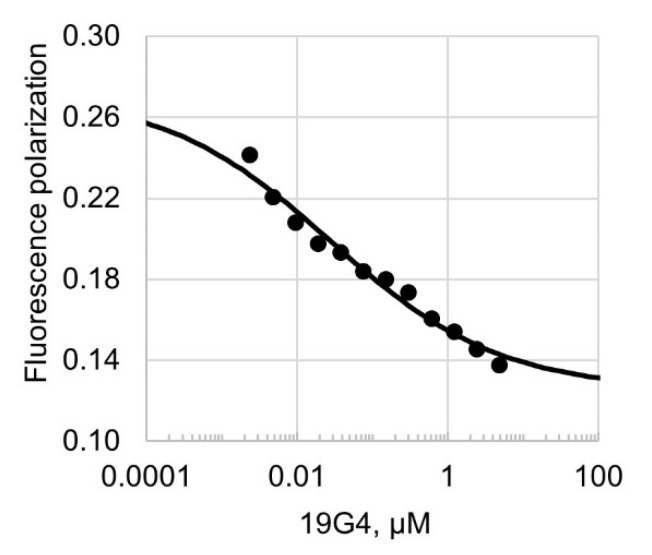
Competitive displacement curve of FAM-labeled 30Tf/30Bf (10 nM) from its preformed complex with Dnmt3a-CD by G4-containing unlabeled 19G4. The amount of displaced 30Tf/30Bf was assessed by fluorescence polarization assay; the measurements were carried out in buffer A in the presence of 100 µM AdoHcy.

In the second series of experiments, fluorescence polarization assay was used to examine the competitive displacement of the FAM-labeled 30Tf/30Bf from the preformed Dnmt3a-CD•30Tf/30Bf complex with unlabeled 19G4 (Figure 6). 

As can be seen, the fluorescence polarization value decreased with increasing 19G4 concentration, which suggests that the G4-containing oligonucleotide displaced the cognate Dnmt3a-CD substrate from the protein•DNA complex. The calculated half-maximal effective concentration (EC_50_) of 19G4 was 26 ± 1 nM.

### 2.4. Effect of G4 Structures on Methylation by MTase Dnmt3a-CD

**Figure 7:**
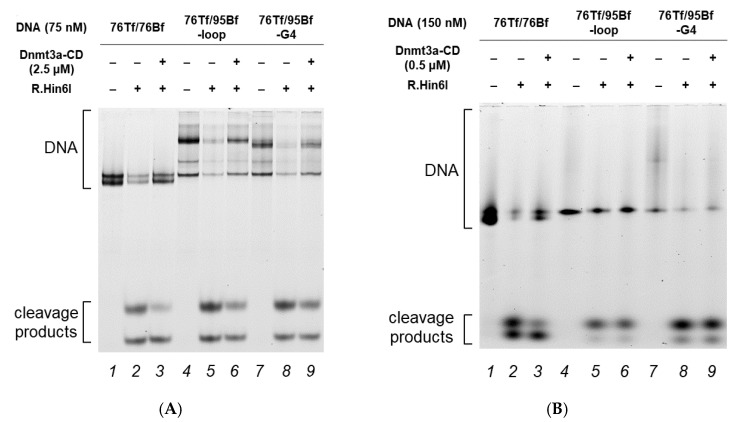
Effect of G4 structures on DNA methylation by Dnmt3a-CD. Cleavage of DNA duplexes by R.Hin6I after their methylation by Dnmt3a-CD. (**A**) 2.5 μM Dnmt3a-CD, 75 nM DNA, (**B**) 0.5 μM Dnmt3a-CD, 150 nM DNA. The reaction mixtures were analyzed in a 10% polyacrylamide gel containing 7 M urea.

In the first series of experiments, the methylation activity of Dnmt3a-CD toward 76Tf/76Bf, 76Tf/95Bf-loop, and 76Tf/95Bf-G4 was examined. In our previously developed assay, DNA duplexes were methylated with Dnmt3a-CD at various concentrations and then digested with the R.Hin6I endonuclease, which cleaves unmethylated R.Hin6I recognition site [29], overlapping with one of the CpG sites located in the duplex flanks. The reaction mixtures were resolved by polyacrylamide gel electrophoresis under denaturing conditions (Figure 7), and the extent of methylation was calculated from the ratio of the combined fluorescence intensity of 8- and 10-nt cleavage products to the total DNA fluorescence intensity (Table 2). 

When 2.5 μM Dnmt3a-CD was used (Dnmt3a-CD to DNA ratio = 33:1), there was no significant difference in the extent of methylation among all DNA duplexes (Table 2). However, when the enzyme concentration was reduced to 0.5 μM (Dnmt3a-CD to DNA ratio = 3:1), only the perfect DNA duplex 76Tf/76Bf underwent effective methylation. Presumably, the inhibition of Dnmt3a-CD observed at a concentration of 0.5 μM is associated with the binding of a significant amount of the enzyme to noncanonical DNA structures. In the case of 2.5 μM Dnmt3a-CD, the number of the enzyme’s molecules not bound with the G4 structure is sufficient for effective methylation.

**Figure 8:**
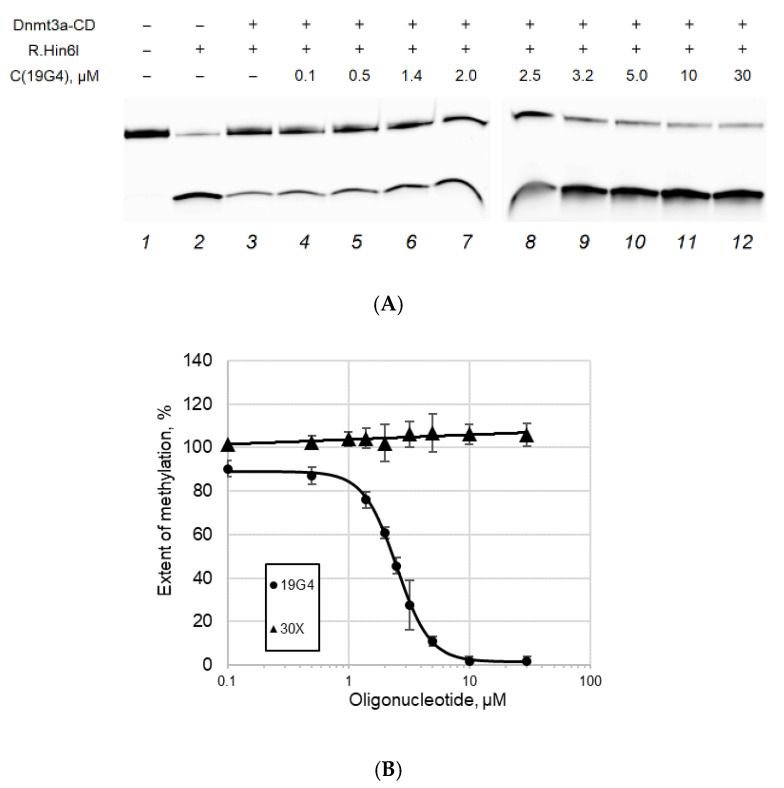
Inhibition of the Dnmt3a-CD-mediated methylation of DNA duplex 30Tf/30Bf (300 nM) by G4-containing oligonucleotide 19G4. Control: a 30-nt oligonucleotide lacking G4 (30X). (**A**) Endonuclease Hin6I-induced cleavage of 30Tf/30Bf after its methylation with 2 μM Dnmt3a-CD in buffer A in the presence of 25 μM AdoMet and 19G4 (concentrations are indicated above the gel lanes). The reaction mixture was analyzed by electrophoresis in 20% polyacrylamide gel containing 7 M urea. (**B**) Extent of methylation as a function of 19G4 or 30X concentration. The standard deviations of three independent measurements are presented.

In the second series of the experiments, we assessed whether the G4 structures could inhibit the activity of Dnmt3a-CD toward 30Tf/30Bf as a cognate substrate. The extent of methylation was calculated as described above. In the absence of 19G4, the 30Tf/30Bf duplex is almost completely methylated (Figure 8). As the concentration of 19G4 in the mixture was raised, the extent of methylation of 30Tf/30Bf decreased, implying the inhibition of Dnmt3a-CD. The dependence of methylation extent on the 19G4 concentration (Figure 8B) was used to calculate the IC_50_: 2.5 ± 0.1 μM. On the contrary, even in the presence of a large excess of an oligonucleotide lacking G4 (30X), no inhibition of MTase activity was observed.

Thus, the effect of G4—either alone as 19G4 (isolated G4) or in a context of double-stranded DNA—on the methylation activity of Dnmt3a-CD was successfully demonstrated experimentally.

## 3. Discussion

Over the past few decades, DNA G4s have been extensively studied in aspects ranging from G4 conformations and biophysical properties to biological functions in normal and pathological cells and determination of the role of G4s in complex biological processes [12,32]. However, the full extent of G4 functions in cells remains to be determined, as well as the scale to which they are beneficial (regulation of gene expression, removal of negative DNA supercoiling, etc.) or detrimental (contribution to genome instability) to cellular processes [33,34]. It was shown that G4s have a diverse effect on the functioning of the main cellular proteins and enzymes interfering with key biological processes. Here, the first steps were taken to establish a relationship between G4 formation and the DNA methylation machinery. We focused on the G4 effect on the function of the mammalian DNA methyltransferase Dnmt3a, which has not yet been investigated in this aspect. The catalytic activity of this enzyme was examined in vitro using an isolated G4 structure (19G4) or G4 embedded in DNA duplex (76Tf/95Bf-G4) (Figure 2B,C). The advantage of the latter model is the possibility to (i) easily vary the number of methylated CpG sites and the distance between them and the G4 structure, and (ii) focus on the methylation of specific CpG sites. The DNA duplex 76Tf/95Bf-G4 was employed to mimic the G4s formed in genomic DNA, in particular, in the promoter regions of oncogenes (Figure 2A) [28]. In this model, the analyzed CpG site is located 28 bp away from G4 (Figure 2C). Since the G4 folding topology and thermal stability of both the G4 and duplex domains of the 76Tf/95Bf-G4 and 76Tf/95Bf-loop were analyzed previously using various biophysical and biochemical techniques [28], we analyzed the secondary structure of 19G4; as shown by CD and UV spectroscopy data, 19G4 folds into a stable G4 structure with a parallel arrangement of G-tracts under the Dnmt3a operating conditions (Figure 3).

An important point considered in our work is determining the role of individual Dnmt3a2 domains in an enzyme interaction with G4. We selected the C-terminal catalytic domain, Dnmt3a-CD, which is known to interact with DNA [19], as well as the PWWP domain, which is a part of the N-terminal regulatory region of the enzyme [23] (Figure 1). PWWP was recently reported to also interact with DNA, albeit with *K_d_* an order of magnitude weaker (3.5 µM [26]) as compared to Dnmt3a-CD (*K_d_* 0.2 µM [35,36]). As part of our study, model recombinant proteins were obtained: full-length murine AIR-Dnmt3a2 with substitutions in the ADD domain to remove autoinhibition, as well as catalytic (Dnmt3a-CD) and regulatory (PWWP) domains.

### 3.1. Dnmt3a-CD, Unlike the PWWP Domain, Effectively Binds to G4 Structures

First, EMSA revealed an effective complex formation between AIR-Dnmt3a2 and 19G4f (Figure 4A, lanes 8 and 9). This result is consistent with surface plasmon resonance data obtained by Cree et al. [17] on the binding of recombinant human DNMT3A to G4-containing oligonucleotides derived from promoters of various human genes (*CDKN1C*, *c-MYC*, and others). Of note, G4s studied by the authors had different G4 motifs compared to 19G4f. Thus, it is reasonable to assume that Dnmt3a is able to bind to quadruplexes embedded in the DNA double helix, regardless of their base composition and G4 scaffold. 

Next, the comparative binding of 19G4f to the catalytic and regulatory domains, Dnmt3a-CD and PWWP, respectively, was analyzed. According to EMSA data, the binding extent of the G4-containing oligonucleotide depended on an enzyme concentration, rising from ~25% to almost 100% with an increase in Dnmt3a-CD concentration from 1 to 4.5 μM (Figure 4A,E). The PWWP domain formed a much weaker complex with 19G4f (<10%), even when the PWWP concentration was higher than that of Dnmt3a-CD. Binding of 19G4f to Dnmt3a-CD or PWWP was then quantified by the fluorescence polarization technique: the *K_d_* of the complex between 19G4f and Dnmt3a-CD proved to be 30 ± 7 nM (Table 1). *K_d_* of the 19G4f•PWWP complex was found to be an order of magnitude weaker, 390 ± 130 nM (Table 1). It can be concluded that Dnmt3a-CD, rather than the PWWP domain, is the AIR-Dnmt3a2 region that effectively interacts with the parallel G4 structure. The efficiency of 19G4f binding to 1 μM Dnmt3a-CD was of the same order of magnitude (~25%) as that of full-length AIR-Dnmt3a2 at the same concentration (Figure 4E). In contrast, the catalytic domain of maintenance DNMT1 probably is not involved in G4 binding [18].

Our findings have been supported by competitive displacement experiments. Thus, unlabeled oligonucleotide 19G4 effectively displaced the DNA substrate, 30Tf/30Bf, from its preformed complex with Dnmt3a-CD (Figure 6), suggesting that the 19G4 binding to Dnmt3a-CD occurs on the DNA-binding surface of the Dnmt3a tetramer. Prokaryotic CpG-recognizing MTase M.SssI, which shares 10 key sequence motifs with Dnmt3a-CD, forms a much weaker complex with 19G4f (Figure 4A, lanes 6 and 7; Figure 5A). These observations may be explained by variations in the structure of DNA complexes with M.SssI [37] and Dnmt3a-CD [19], in particular, in the organization of the enzyme’s binding site on the DNA substrate. The ability of Dnmt3a-CD to tetramerize is crucial for the assembly of DNA-binding surfaces [38], which appear to be more attractive for G4 structures than the DNA-binding surface of monomeric M.SssI.

Binding of 76Tf/95Bf-G4 to AIR-Dnmt3a2 and its domains follows the same trend as observed for oligonucleotide 19G4f (Figure 4B–E). AIR-Dnmt3a2 and Dnmt3a-CD bind to this DNA duplex stronger than PWWP does. Consequently, Dnmt3a-CD seems to be the domain capable of binding to G4 stabilized in the duplex context. Based on these data, we hypothesize that the G4 formation within gene promoter regions is unlikely to alter the interaction of the PWWP domain with a nucleosome (namely, with the H3K36me2/3 tail of a histone and with DNA).

### 3.2. G4-Containing DNA Duplex Shows Improved Binding and Impaired Methylation by Dnmt3a-CD

According to the EMSA data, insertion of G4 into the DNA duplex (76Tf/95Bf-G4) significantly affected the DNA binding affinity to Dnmt3a-CD (Figure 4B–E). Accordingly, the strong binding of the enzyme to the isolated quadruplex in 19G4f was observed by EMSA and fluorescence polarization assay. The methylation efficiency of 76Tf/95Bf-G4 with 0.5 μM Dnmt3a-CD (MTase:DNA ratio of 3:1) was markedly lower than that of the control DNA. It can be assumed that the MTase is able to bind both the G4 structure and the CpG-bearing duplex flanks of the substrate. The noncanonical G4 structure is likely to compete for binding to the CpG site or preclude the formation of filament-like enzyme oligomers on DNA [20], leading to reduced methylation efficiency. Dnmt3a-CD tends to multimerize on long DNA duplexes, which leads to a non-linear increase in protein activity, and this effect becomes more pronounced as the protein concentration increases [35]. The results obtained from our EMSA, fluorescence polarization and methylation experiments showed that the DNA duplexes 76Tf/95Bf-G4 and 76Tf/95Bf-loop exhibited similar behavior. These findings support the hypothesis that the presence of a loop may also lead to a disruption of Dnmt3a-CD filaments.

Thus, the presence of a G4 insert or an unstructured loop in double-stranded DNA reduces the extent of Dnmt3a-CD-mediated CpG methylation, although a local distortion of regular DNA structure (G4 or loop) is located at a distance of 28 bp from the CpG site in question. This influence can manifest itself in two ways. First, most likely, Dnmt3a-CD is sequestered on G4 structure forming a strong protein•DNA complex (Figure 5, Table 1). The possibility of such sequestration of MTase on DNA has been documented for MTase DNMT1 [18]. Second, G4 or a DNA loop can create steric hindrances for the oligomerization of the Dnmt3a-CD tetramers during its binding to DNA. Notably, the ability of G4 structures to serve as an obstacle for the functioning of mammalian CpG methyltransferases is considered for the first time. To clarify the mechanisms underlying the effect of G4 structures on DNA methylation, further improvement of the model systems is required, in particular, varying the distance between CpG sites and G4 structures in the DNA sequences mimicking promoter regions of various genes.

### 3.3. G4-Forming Oligonucleotide 19G4 Inhibits Dnmt3a-CD Activity

Based on competitive displacement experiments, Dnmt3a-CD-mediated methylation of cognate substrate, 30Tf/30Bf, was shown to be significantly inhibited in the presence of 19G4 (Figure 8). The effect of the G4 on methylation by Dnmt3a (IC_50_ = 2.5 ± 0.1 µM) was comparable to that of the minor-groove–specific and intercalating DNA ligands: olivomycin A [39] and curaxin CBL0137 [40]. In this regard, we suggest that G4-forming oligonucleotides can serve as the basis for designing MTase-targeting aptamers. G4-based MTase inhibitors may be beneficial in treating various cancers, such as acute myeloid leukemia, characterized by genome hypermethylation and the presence of mutant Dnmt3a proteins [41]. 

## 4. Materials and Methods

### 4.1. Reagents 

All oligonucleotides (Figure 2) (synthesized via standard phosphoramidite chemistry and purified by high-pressure liquid chromatography by Evrogen, Moscow, Russia, and Syntol, Moscow, Russia) were used without further purification. Some of the oligonucleotides contained a fluorescent dye indicated as “f”: either FAM (Figure 2) or TAMRA. Concentrations of oligonucleotides were determined spectrophotometrically at 260 nm as described previously [28]; the extinction coefficients were derived from the nearest-neighbor data (https://www.idtdna.com/calc/analyzer (accessed on 21 January 2022); https://web.expasy.org/protparam/ (accessed on 21 January 2022)). AdoMet and AdoHcy were obtained from Sigma (Burlington, MA, USA). The following buffer solutions were used: 20 mM HEPES-NaOH (pH 7.5), 100 mM KCl, 1 mM EDTA, and 1 mM 1,4-dithiothreitol (buffer A), 10 mM Tris-HCl (pH 7.3), 70 mM NaCl, and 2.5 mM KCl (buffer B), and 90 mM Tris, 90 mM H_3_BO_3_ (pH 8.3), and 2 mM EDTA (buffer C). To assemble G4 and DNA duplex structures, oligonucleotides or equimolar mixtures of complementary (or partly complementary) oligonucleotides were annealed in buffer A or B by heating at 90–95 °C for 3 min and slowly cooling to 25 °C. 

### 4.2. Enzymes and Proteins 

Restriction endonuclease Hin6I was purchased from SibEnzyme (Novosibirsk, Russia). Prokaryotic MTase SssI was purified as described previously [42]. To isolate a mutant full-length AIR-Dnmt3a2 enzyme with removed autoinhibition and the Dnmt3a catalytic domain (Dnmt3a-CD), *Escherichia coli* BL21(DE3) cells were transformed with plasmid pET-28a(+) carrying the gene encoding one of the corresponding murine proteins with an N-terminal 6 × His tag. Two amino acid substitutions in AIR-Dnmt3a2 were implemented by site-directed mutagenesis according to the QuikChange protocol (Agilent, Santa Clara, CA, USA). The presence of the introduced mutations was verified by Sanger sequencing by Evrogen (Moscow, Russia). The PWWP domain was produced in *E. coli* BL21(DE3) cells transformed with the pGEX-6P-2 plasmid harboring the gene coding for the PWWP domain of murine Dnmt3a (amino acid residues 279 to 420 [23]) fused with *Schistosoma japonicum* glutathione S-transferase (GST) at the N-terminus. Between the sequences encoding GST and PWWP, there is a sequence coding for a linker: SDLEVLFQGPLGS. Dnmt3a-CD and AIR-Dnmt3a2 were isolated and purified by metal affinity chromatography on a Co^2+^-containing TALON^®^ resin (GE Healthcare, Chicago, IL, USA) [43]. The PWWP domain was purified by affinity chromatography on the Glutathione Sepharose 4B resin (GE Healthcare, Chicago, IL, USA). The purity of the protein samples was evaluated by electrophoresis in a 12% polyacrylamide gel containing sodium dodecyl sulfate [44]. The protein concentrations were determined by the Bradford assay per protein monomer. The resulting proteins were aliquoted and stored at −80 °C.

### 4.3. Circular Dichroism Measurements

Oligonucleotide 19G4 (Table 1) was annealed in buffer A containing 100 mM KCl to enable G4 formation. CD spectra were recorded in a quartz cuvette of 10 mm optical path length at room temperature or between 10 and 90 °C in ~5 °C increment at an average heating rate of 0.5 °C/min on a Chirascan CD spectrometer (Applied Photophysics Ltd., Leatherhead, UK). The DNA concentration was chosen to attain absorption of 0.6–0.8 at 260 nm, which gives an optimal signal-to-noise ratio. The measurements were performed in the 220–360 nm wavelength range at a scanning speed of 30 nm/min and a signal averaging time of 2 s with constant flow of dry nitrogen. The CD spectra were baseline-corrected for signal contributions caused by the buffer and plotted as molar dichroism per oligonucleotide strand against wavelength. The spectra were processed with the Origin 8.0 software (OriginLab, Northampton, MA, USA) using the Savitzky–Golay filter. 

### 4.4. UV Spectroscopy Melting of G4 Structure

The procedure of sample preparation for UV spectroscopy measurements was the same as that for the CD experiments. Absorbance-versus-temperature profile of 19G4 (at ~3 µM concentration per oligonucleotide strand) was recorded in buffer B in a 600 μL quartz microcuvette (Hellma Analytics, Müllheim, Germany) with an optical path length of 10 mm on a double-beam Hitachi U-2900 UV/visible spectrophotometer (Hitachi, Tokyo, Japan) equipped with a Hitachi thermoelectric controller. Changes in absorbance were monitored between 25 and 85 °C at 295 nm at a heating rate of 0.5 °C/min. *T*_m_ was estimated from an extremum value of the first derivative of the fitted curve for data smoothed with the Savitzky–Golay filter.

### 4.5. DNA Binding Measurements

#### 4.5.1. Electrophoretic Mobility Shift Assay 

EMSA was employed to analyze the complex formation of AIR-Dnmt3a2 and its functional domains with DNA. FAM- or TAMRA-labeled DNA fragments 19G4f, 22(GAGT)_4_f, 76Tf/76Bf, 76Tf/95Bf-G4, or 76Tf/95Bf-loop (Figure 2 and Figure S2) (60 nM) were incubated with different concentrations of AIR-Dnmt3a2, Dnmt3a-CD, PWWP, or M.SssI in buffer A containing 6% glycerol and 100 µM AdoHcy at 25 °C for 30 min. Unbound DNA and protein•DNA complexes were separated by electrophoresis in a 3% polyacrylamide gel under non-denaturing conditions at 4 °C in buffer C. Relative intensity of the DNA bands on electropherograms obtained with Typhoon FLA 9500 scanner (GE Healthcare, Chicago, IL, USA) was evaluated using the GelQuantNET 1.7.8 software provided by biochemlabsolutions.com (San Francisco, CA, USA). The fluorescence intensities of unbound (*I_unbound_*), total (*I_total_*) and bound (*I_bound_* = *I_total_* − *I_unbound_*) DNA were determined. *I_bound_* represented the fluorescence intensity of DNA in protein•DNA complexes, including those that failed to enter the gel. The extent of proteins binding to DNA was determined using the equation:

$$Extent\;of\;binding=\frac{I_{bound}}{I_{total}}\;\times \;100\%$$

#### 4.5.2. Fluorescence Polarization Assay 

The binding of Dnmt3a domains to 19G4f, 30Tf, or 30Tf/30Bf was investigated by a fluorescence polarization assay via direct titration of FAM-labeled DNA duplexes with MTase domains in the presence of AdoHcy (100 µM) as described before [40]. Various amounts of a protein under investigation were added to a mixture containing 10 nM DNA in buffer A, and fluorescence polarization (*P*) was measured using a Cary Eclipse spectrofluorometer (Varian, Palo Alto, CA, USA) with excitation at 495 nm and emission at 520 nm at 25 °C in 120 µL cuvettes with a 1 cm pathlength. *P* was calculated according to the equation *P* = (*I_v_* − *G*·*I_h_*)/(*I_v_* + *I_h_*), where *I_v_* and *I_h_* are vertical and horizontal components of the emitted light, respectively, and *G* is a correction factor that was measured once before each experiment. The experimental data are presented as a dependence of *P* on the total protein concentration. The titration curves of each DNA duplex with the MTase were obtained in at least two technical replicates. Dissociation constants (*K_d_*) of the complexes were obtained by the fitting of the observed *P* dependences on the protein concentration to a logistic equation using the OriginPro 8.0 software (OriginLab, Northampton, MA, USA):

$$P=\frac{P_{MAX}-P_{MIN}}{1+{\left(\frac{C_{DNA}}{K_{d}}\right)}^{n}}+P_{MIN},$$

where *P_MIN_* and *P_MAX_* are initial and final fluorescence polarization, respectively; *n* is a slope factor; and *C_DNA_* is DNA concentration.

The competition of oligonucleotide 19G4 for complex formation with Dnmt3a-CD was assessed in buffer A containing 100 mM KCl by the addition of various amounts of competitor oligonucleotide to a preformed complex of Dnmt3a-CD (100 nM) with FAM-labeled DNA duplex 30Tf/30Bf (10 nM) as a cognate MTase substrate and by measuring *P* values as described above. The ratio of bound DNA to total DNA in the initial complex determined from the binding curve of 30Tf/30Bf and Dnmt3a-CD was ~0.6. Half-maximal effective concentration (EC_50_) was calculated by fitting the obtained dependences of *P* on the concentration of a DNA substrate to the dose–response equation.

### 4.6. Methylation Assay

#### 4.6.1. Methylation of Double-Stranded DNA Substrates, including Those Containing G4 Structure

MTase activity was analyzed by protection of the methylated DNA from cleavage by restriction endonuclease Hin6I [29]. TAMRA-labeled DNA duplex 76Tf/76Bf, 76Tf/95Bf-G4, or 76Tf/95Bf-loop (75 or 150 nM) was incubated in buffer A for 2 h at 37 °C at various concentrations of Dnmt3a-CD (0.5 or 2.5 μM) in the presence of 25 μM AdoMet. After methylation, the samples were treated with proteinase K (100 µg/mL) for 30 min at 55 °C to inactivate Dnmt3a-CD and facilitate the entry of the DNA strands into a polyacrylamide gel. Proteinase K was subsequently inactivated via heating of the samples to 95 °C followed by slow cooling to 25 °C. Next, the mixtures were incubated with 1 U of R.Hin6I for 1 h at 37 °C in the presence of 3 mM MgCl_2_, analyzed by electrophoresis in a 10% polyacrylamide gel with 7 M urea and visualized with the Typhoon FLA 9500 scanner (GE Healthcare, Chicago, IL, USA). The fluorescence intensities of intact DNA and cleavage products were determined using the GelQuantNET 1.7.8 software provided by biochemlabsolutions.com (San Francisco, CA, USA). The extent of DNA cleavage (*w*) was calculated as a ratio of fluorescence intensity of the cleaved DNA to the total fluorescence intensity of the intact and cleaved DNA. The extent of methylation (*M*) was calculated using the equation:

$$M=\frac{w_{0\;}-w_{Dnmt3a}}{w_{0}},$$

where *w*_0_ and *w*_*Dnmt*3*a*_ are the extent of DNA cleavage before and after methylation by Dnmt3a-CD, respectively. When calculating the extent of cleavage, we employed total fluorescence intensities of the initial DNA duplexes, because under denaturing conditions, the strands do not separate completely due to high stability of the extended DNA double helix. Intensities of 8- and 10-nt cleavage products were summed during the calculation.

#### 4.6.2. Effect of 19G4 on Methylation of 30Tf/30Bf by Dnmt3a-CD 

FAM-labeled DNA duplex 30Tf/30Bf (300 nM) containing the CpG methylation site within the R.Hin6I recognition site (Figure 2D) was incubated in buffer A for 1 h at room temperature at various concentrations of oligonucleotide 19G4. After that, the duplex was methylated with 2 µM Dnmt3a-CD in the presence of 25 µM AdoMet for 1.5 h at 37 °C. Then, 3 mM MgCl_2_ was introduced into the mixtures, and the DNA duplex was digested with 2 U of R.Hin6I for 1 h at 37 °C. In control mixtures, the cleavage was carried out without prior methylation. The extent of methylation was calculated as described above. IC_50_ values were calculated via fitting of the extent of 30Tf/30Bf duplex methylation in the dependence on 19G4 concentration to the following equation using OriginPro 8.0 software:

$$M=M_{MIN}+\frac{M_{MAX}-M_{MIN}}{1+{\left(\frac{C_{DNA}}{IC_{50}}\right)}^{p}},$$

where *M* is the extent of methylation, *M_MIN_* and *M_MAX_* are lower and upper asymptotes, *C_DNA_* is concentration of 19G4f, and *p* is a slope factor.

## 5. Conclusions

Here, we provided experimental evidence for a communication between noncanonical G4 structures and DNA methylation status. Two types of G4 models were used: a single-stranded 19G4 oligonucleotide containing a G4-forming insert and a double-stranded DNA in which the G4 structure was stabilized in a quasi-genomic double-stranded context containing CpG sites (Table 1). The mammalian methyltransferase Dnmt3a was found to recognize and efficiently bind G4 in both DNA models. These findings are in agreement with the proposed G4 contribution to DNA binding to various chromatin-remodeling factors, transcription factors, and DNA-modifying enzymes [7,16]. It has been shown for the first time that the Dnmt3a binding to G4 is mediated predominantly by the catalytic domain of the enzyme. On the contrary, the PWWP regulatory domain forms a much weaker complex with G4 structure. Importantly, a quadruplex-bearing 19G4 oligonucleotide displaced a DNA duplex substrate from its complex with the catalytic domain of the Dnmt3a enzyme, its methylation activity being suppressed. Moreover, the direct inhibitory effect of G4 on the Dnmt3a-mediated methylation was experimentally detected for the DNA duplex (76Tf/95Bf-G4) with the G4 structure located 28 bp away from the CpG site under consideration. Further research is needed to elucidate the molecular mechanisms that govern G4-mediated processes of epigenetic regulation and to characterize new aspects of G4 biology.

## Figures and Tables

**Table 1:** Dissociation constants (*K*_d_) of 19G4f, 30Tf or 30Tf/30Bf complexes with each protein under investigation.

Protein	*K_d_* *, nM		
	19G4f	Controls	
		30Tf	30Tf/30Bf
Dnmt3a-CD	30 ± 7	200 ± 30	80 ± 20
PWWP	390 ± 130	-	-
M.SssI	320 ± 150	-	-

* Standard deviation of two independent measurements.

**Table 2:** Extent of methylation of DNA duplexes at different Dnmt3a-CD:DNA ratios.

DNA Duplex	Extent of DNA Methylation *, %	
	Dnmt3a-CD:DNA Ratio	
	33:1	3:1
76Tf/76Bf	56 ± 7	42 ± 5
76Tf/95Bf-G4	48 ± 8	8 ± 3
76Tf/95Bf-loop	50 ± 5	12 ± 3

* Standard deviation of at least three independent measurements.

## Data Availability

Not applicable.

## Supplementary Materials

The following supporting information can be downloaded at: https://www.mdpi.com/article/10.3390/ijms231810226/s1.
