# Supplementary material for: Impact of G-Quadruplex Structures on Methylation of Model Substrates by DNA Methyltransferase Dnmt3a
Source: Int J Mol Sci. 2022 Sep 6;23(18):10226. doi: 10.3390/ijms231810226 (PMC9499004; doi:10.3390/ijms231810226)
Supplement: Supplementary file 1 [file ijms-23-10226-s001.zip › ijms-1868017-supplementary.pdf]

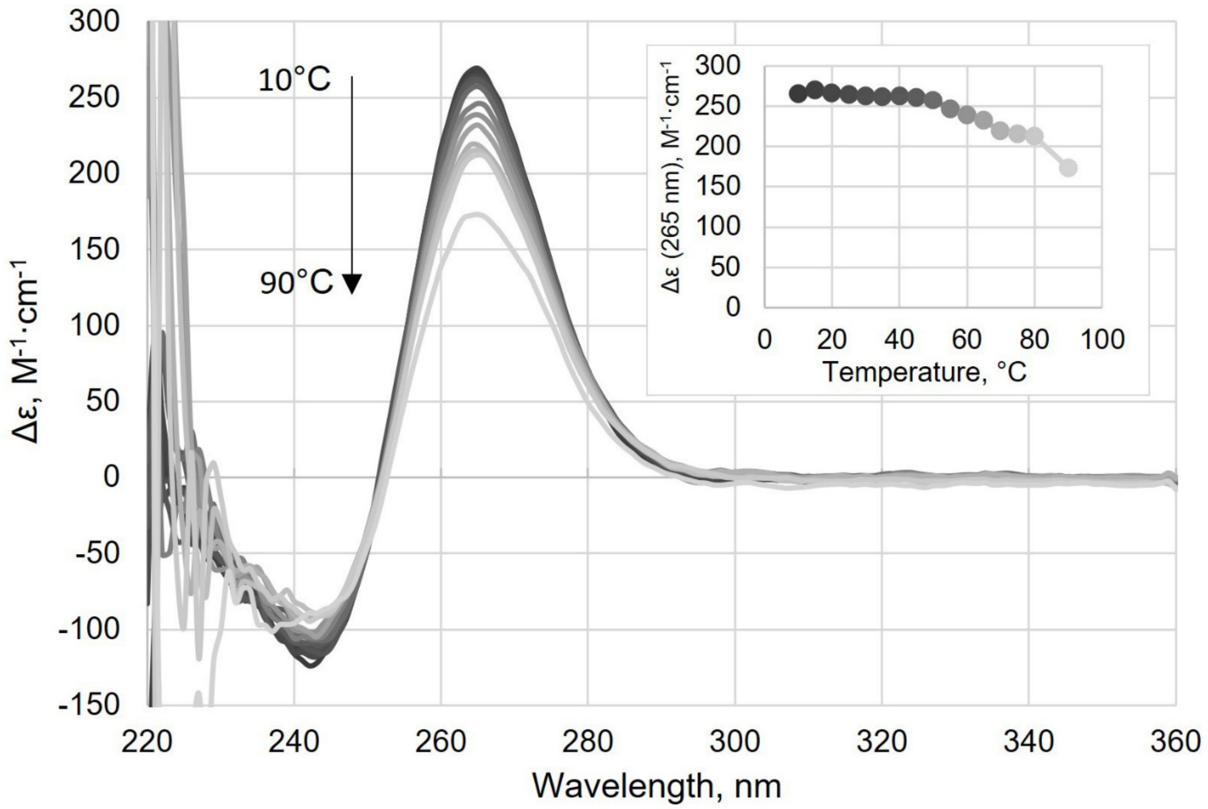


**Figure S1.** The CD spectra of the oligonucleotide 19G4 recorded in buffer A containing 100 mM KCl at different temperatures (~2 μM oligonucleotide concentration). The arrow indicates an increase in temperature from 10 to 90 °C in 5 °C increments; gray lines of various shades show the CD spectra at different temperatures, rising in the direction of the arrows. (**Inset**) CD-monitored melting profiles at 265 nm.

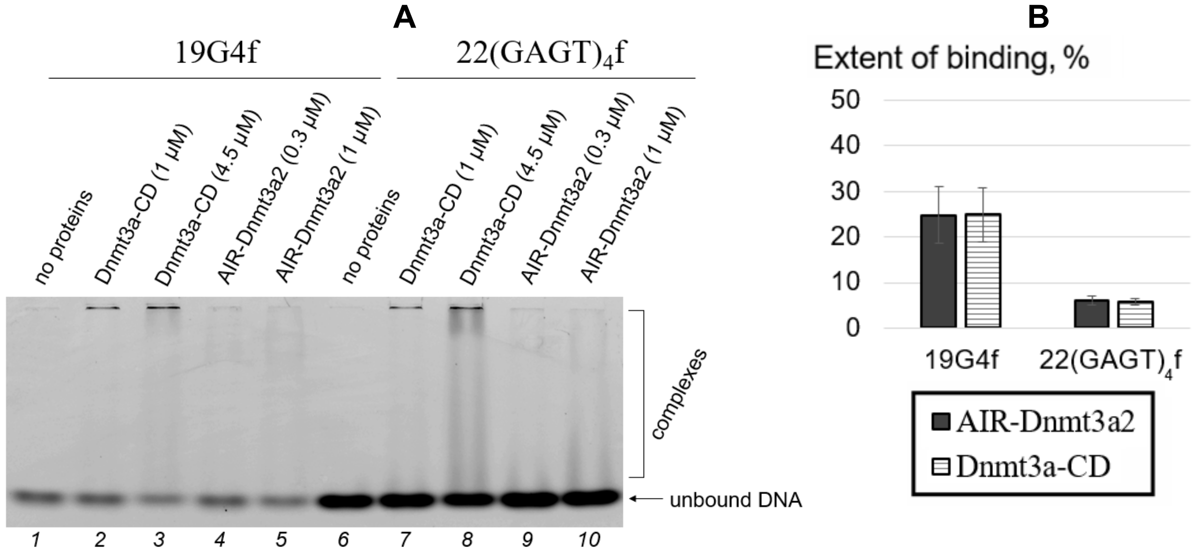


**Figure S2.** Binding affinity of full-length AIR-Dnmt3a2 and its catalytic domain Dnmt3a-CD to 19G4f and 22(GAGT)$_4$f using EMSA. (**A**) Protein•DNA complex formation recorded by electrophoresis in 3% non-denaturing polyacrylamide gel. Zones of protein•DNA complexes are indicated by parenthesis; the arrow on the right indicates the position of the unbound DNA. 19G4f (lanes 1-5) or 22(GAGT)$_4$f (lanes 6-10) were incubated for 30 min in buffer A containing 100 mM KCl with AIR-Dnmt3a2 or Dnmt3a-CD. (**B**) Extent of DNA binding to 1 μM AIR-Dnmt3a2 or Dnmt3a-CD calculated as described in Section 4.5.1.

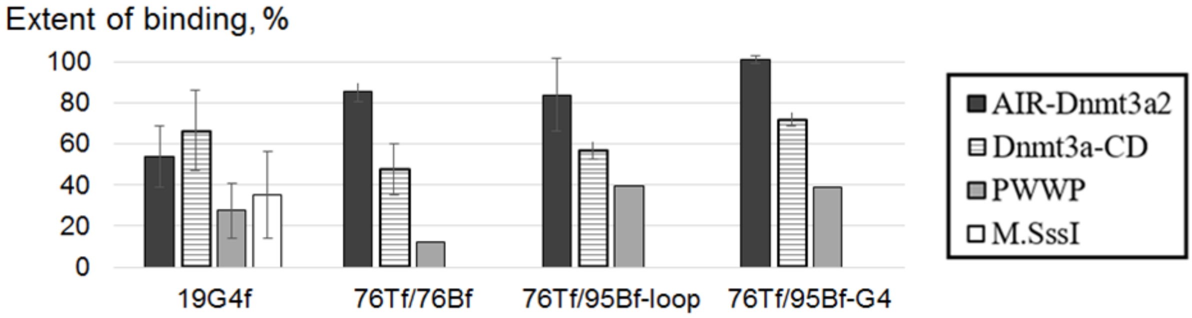


**Figure S3.** The extent of DNA binding to AIR-Dnmt3a2 (1 μM), Dnmt3a-CD (1 μM), PWWP (3 μM), or M.SssI (2 μM) calculated from EMSA data (Figure 4A-D). The calculation was based on the decrease in the fluorescence intensity of unbound DNA upon the protein addition:

$$Extent\ of\ binding = \frac{(I_{initialDNA} - I_{unboundDNA})}{I_{initialDNA}} * 100\%,$$

where $I_{initial\ DNA}$ and $I_{unbound}$ are the fluorescence intensities of unbound DNA in the lanes before and after the addition of proteins, respectively. The standard deviations of two independent measurements are indicated.

*Annotation to Figure S3*

To validate the DNA-MTase binding affinity estimated in Section 2.3.1 (Figure 4E), we added an alternative calculation approach based on the reduction in unbound DNA fluorescence compared to the initial DNA fluorescence intensity (Figure S3). For DNA duplexes 76Tf/76Bf, 76Tf/95Bf-G4 and 76Tf/95Bf-loop, the extents of their binding to proteins were almost equal to those given in Figure 4E. As for the 19G4f oligonucleotide, the same trend persists: the extent of 19G4f binding to the MTases AIR-Dnmt3a2 and Dnmt3a-CD was close in value, while the binding of the oligonucleotide to the PWWP domain was significantly worse.
